# *Bombyx mori* C-Type Lectin (BmIML-2) Inhibits the Proliferation of *B. mori* Nucleopolyhedrovirus (BmNPV) through Involvement in Apoptosis

**DOI:** 10.3390/ijms23158369

**Published:** 2022-07-28

**Authors:** Xianghan Mei, Chun Li, Peilin Peng, Jue Wang, Enxi He, Zhiyong Qiu, Dingguo Xia, Qiaoling Zhao, Dongxu Shen

**Affiliations:** 1 Jiangsu Key Laboratory of Sericultural Biology and Biotechnology, School of Biotechnology, Jiangsu University of Science and Technology, Zhenjiang 212018, China; xianghanmei@126.com (X.M.); infplichun0320@163.com (C.L.); pengpl0321@163.com (P.P.); wangwangyu163@163.com (J.W.); heenxi1361178044@163.com (E.H.); zyqiu2266@126.com (Z.Q.); xiadingguo@126.com (D.X.); qlzhao302@126.com (Q.Z.); 2 Key Laboratory of Silkworm and Mulberry Genetic Improvement, Ministry of Agriculture and Rural Affairs, Sericultural Research Institute, Chinese Academy of Agricultural Sciences, Zhenjiang 212018, China

**Keywords:** *Bombyx mori*, C-type lectin, *B. mori* nucleopolyhedrovirus, apoptosis

## Abstract

C-type lectins (CTLs) are widely distributed in mammals, insects, and plants, which act as pattern recognition receptors (PRRs) to recognize pathogens and initiate immune responses. In this study, we identified a C-type lectin gene called *BmIML-2* from the silkworm *Bombyx mori*. Its open reading frame (ORF) encodes 314 amino acids, which contain dual tandem C-type lectin-like domain (CTLD). *BmIML-2* is highly expressed in the fat body and is significantly induced at 24 h after BmNPV infection. Moreover, overexpression of *BmIML-2* dramatically inhibited the proliferation of BmNPV, and knockdown assay via siRNA further validated the inhibition of *BmIML-2* on viral proliferation. In addition, transcript level detection of apoptosis-related genes and observation of apoptosis bodies implied that overexpression of *BmIML-2* promoted BmNPV-induced apoptosis. Immunofluorescence analysis indicated that BmIML-2 distributed throughout the cytoplasm and was slightly concentrated in the cell membrane. Taken together, our results suggest that BmIML-2 could inhibit in the proliferation of BmNPV by facilitating cell apoptosis.

## 1. Introduction

The domestic silkworm, *Bombyx mori*, is an important economic insect and model organism of lepidoptera, having important economic and scientific research value [1]. For instance, silk from the silkworm is a biomaterial with excellent biocompatibility [2]. Moreover, the silkworm model is suitable for drug discovery due to its low cost, few ethical concerns, and similar pharmacokinetic and pharmacodynamic properties [3,4]. *B. mori* nucleopolyhedrovirus (BmNPV) is an obligate virus that specially infects insects; the disease caused by BmNPV is extremely contagious and seriously endangers sericulture production around the world [5]. However, the molecular mechanism of BmNPV infection is not yet fully understood. In recent years, studies on the infection mechanism of BmNPV has been in full swing, and many effectors involved in viral invasion have been identified and verified. For instance, several studies have been devoted to elucidating the viral invasion mechanism from transcriptomic or proteomic perspective [6,7]. For example, *B. mori* E3 ubiquitin–protein ligase SINA-like 10 (SINAL10) could initiate viral proliferation, which serves as a binding protein for envelope fusion protein GP64 of BmNPV [8]. Additionally, it has been reported that *B. mori* membrane protein BmREEPa is required for BmNPV to enter into cells by interacting with GP64 [9]. 

Insects’ innate immune system is evolutionarily highly conserved, defending the host from invading pathogenic microorganisms, such as bacteria, fungi, and viruses. The innate immunity is triggered by the recognition of pathogen-associated molecular patterns (PAMPs) by the host itself [10]. In general, the components located on the surface of microbial cell walls, such as lipopolysaccharide, lipoteichoic acid, and peptidoglycan, all belong to the category of PAMPs [11]. In insects, pattern recognition receptors (PRRs) are crucial in the recognition of PAMPs, which include C-type lectins (CTLs), peptidoglycan recognition proteins (PGRPs), β-1,3 glucan recognition protein (βGRP), Gram-negative-bacteria-binding proteins (GNBPs), integrins, hemolin, and apolipophorin Ⅲ (ApoLp-Ⅲ), among others [11,12]. The recognition of invading pathogens by the host itself activates downstream signal transduction pathways and thereby a series of innate immune responses, including the synthesis of antimicrobial peptides (AMPs), and here the melanization is initiated, finally killing and eliminating the pathogens. In *Drosophila melanogaster*, the mutants of IMD pathway are defective for PGRP-LC, and other genes displayed more sensitivity to cricket paralysis virus (CrPV) infection [13]. The expression of *BmPGRP*2-2 could be induced by BmNPV, wherein the protein inhibits PTEN-phosphoinositide 3-kinase (PI3K)/Akt signaling pathway to promote BmNPV replication [14]. 

C-type lectins, as highly conserved calcium-dependent receptors, are defined by one or more C-type lectin-like domains (CTLDs). CTLDs that contain characteristic motifs, such as Glu-Pro-Asn (EPN) motif or Gln-Pro-Asp (QPD) motif, are closely linked with the specificity of carbohydrate binding of CTLs [15]. Up until now, C-type lectins have been identified in many mammals, plants, insects, mollusks, and other multicellular organisms [16]. Mammalian CTLs are known to play important roles in the homeostasis of the immune system, allergic responses, recognition of apoptotic cells and tumors, and complement activation [17,18]. Among the arthropods, CTLs specifically recognize the PAMPs on the surface of microbes, and then trigger the aforementioned immune responses to eliminate the invading pathogens. For instance, *B. mori* CTL5 may be involved in the JAK/STAT pathway and slightly stimulated by *B. mori* cytoplasmic polyhedrosis virus (BmCPV) [19]. In mosquitos, mosGCTL-7 interacts with the envelope protein of the Japanese encephalitis virus (JEV) via N-glycan at N154, and then promotes virus entry into cells [20]. *Aedes aegypti* mosGCTL-1 collaborates with the human CD45 homolog mosPTP-1 to enable attachment and entry of the West Nile virus (WNV) [21]. In *Musca domestica* L, MdCTL1 and MdCTL2 could effectively reduce the infection of Sf 9 cells by *Autographa californica* multicapsid nucleopolyhedrovirus (AcMNPV) and dramatically inhibit the proliferation of influenza H1 N1 virus [22]. In short, the roles of insect C-type lectins in viral infection have been increasingly investigated and reported. However, little is known about the potential internal relationship between C-type lectins with BmNPV infection and replication. 

In the present study, we investigated the functional roles of C-type lectins BmIML-2 in viral infection and replication. At first, bioinformatics analysis indicated that BmIML-2 possesses two tandem CTLD that defined a canonical CTL in insects. The phylogenetic tree analysis showed that BmIML-2 has the closest homologous evolutionary relationship with *B. mori* IML-3 and *Manduca sexta* IML-2. In addition, overexpression assay and siRNA interference-mediated knockdown were conducted. The results of overexpression and knockdown demonstrated that *BmIML-2* acts as a negative regulator in the process of viral infection and replication. Moreover, the results of qRT-PCR revealed that *BmIML-2* may take part in the initiation of apoptosis, thus regulating the expression of several apoptosis-related genes. Furthermore, immunofluorescence assay was performed to analyze the subcellular localization of BmIML-2. In sum, our findings demonstrated that BmIML-2 could inhibit the infection and replication of BmNPV, which may be due to its involvement in the regulation of the apoptotic signal pathway.

## 2. Results

### 2.1. Sequence Characteristics and Bioinformatics Analysis of BmIML-2

**Figure 1:**
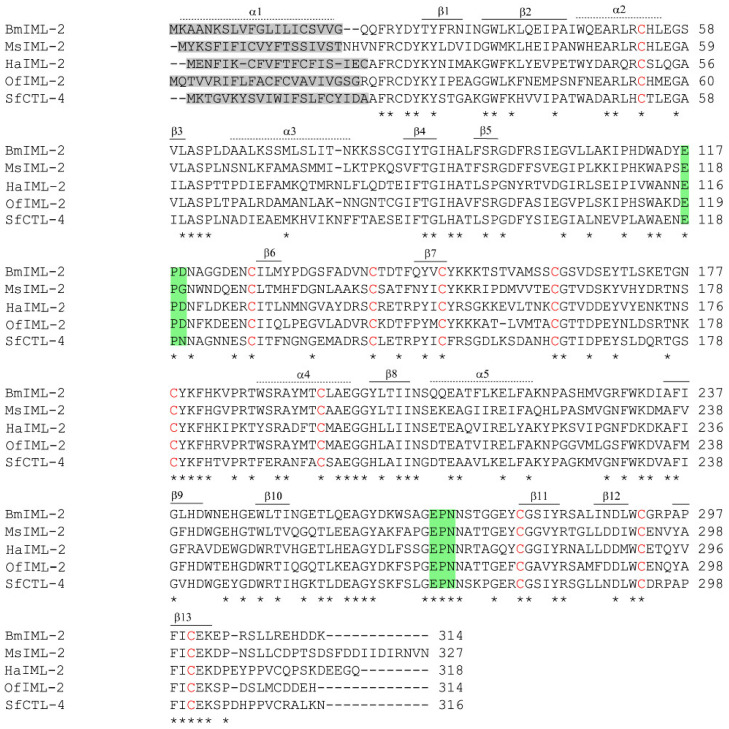
Multiple sequence alignment of *B. mori* IML-2. The predicted signal peptides are shaded with a *gray* background. The conserved cysteine residues in CTLD are shown in *red* font. The *asterisk* below the sequences represents the consistent amino acid residues. The key residues that define sugar-binding specificity are indicated with a *green* background. The secondary structure elements of *B. mori* IML-2 are marked above or below the sequences. The GenBank accession numbers are as follows: BmIML-2, NP_001165396.1; MsIML-2, XP_030038244.1; HaIML-2, ACI32834; OfIML-2, AIR95998.1; SfCTL-4, XP_035441949.1.

**Figure 2:**
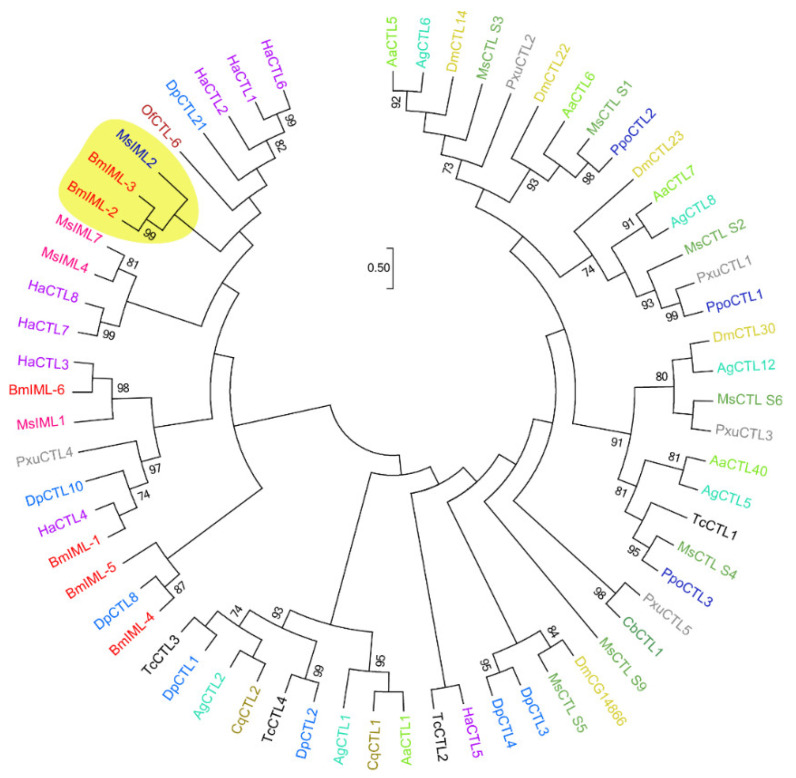
The phylogenetic relationships of *B. mori* IML-2 with other homologous sequences. The branch containing *B. mori* IML-2 shown with a *yellow* background. The numerical values representing bootstrap values only over 70 are marked. The abbreviations are as follows: Aa: *A. aegypti*; Ag: *A. gambiae*; Bm: *B. mori*; Cb: *Cerapachys biroi*; Cq: *Culex quinquefasciat*; Dm: *D. melanogaster*; Dp: *D. plexippus*; Ha: *H. armigera*; Ms: *M. sexta*; Of: *Ostrinia furnacalis*; Ppo: *Papilio polytes*; Pxu: *Papilio xuthus*; Tc: *T. castaneum*.

We identified and characterized the cDNA sequence of *BmIML-2* on the basis of the silkworm genome database. The ORF of *BmIML-2* encodes 314 amino acids that contain a signal peptide of 20 amino acids. The mature protein was predicted with a molecular weight of 35.3 kDa and an isoelectric point of 5.93. As a canonical C-type lectin, the polypeptide sequence of BmIML-2 contains two tandem CTLDs with the EPD and EPN motifs, separately (Figure 1). Moreover, the phylogenetic tree was constructed by using the neighbor-joining method to analyze evolutionary relationships of BmIML-2. As shown in Figure 2, the result indicated that BmIML-2 was tightly grouped with *B. mori* IML-3 and *M. sexta* IML-2 and formed the closest orthologous cluster with *B. mori* IML-3.

### 2.2. Tissue and Induced Expression Pattern of BmIML-2

**Figure 3:**
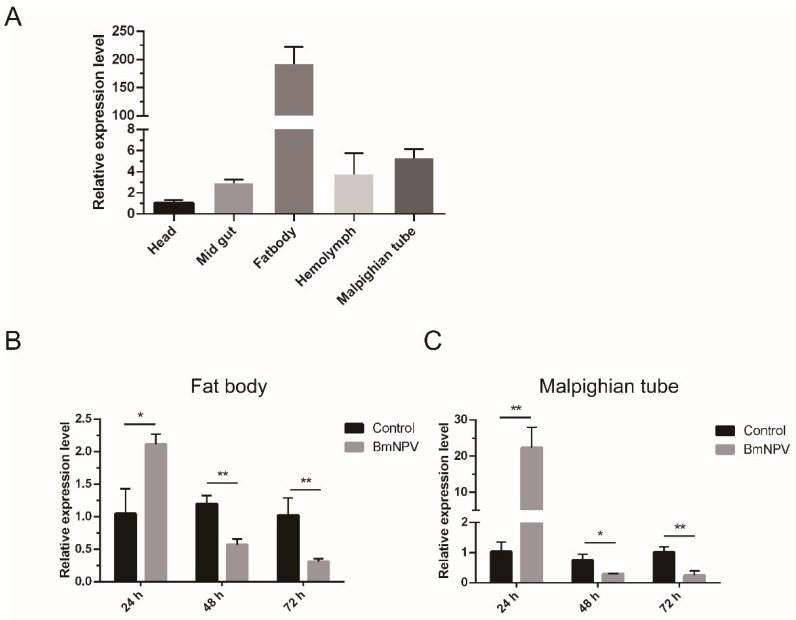
The expression profiles analysis of *B. mori IML-2*. (**A**) The mRNA levels of *BmIML-2* in various tissues. (**B**,**C**) Inducible expression profiles of *BmIML-2* in fat body and malpighian tube upon BmNPV infection. The RNA samples of different tissues were extracted individually as described above. Then, the prepared RNA samples were converted into cDNA for qRT-PCR assay. The *B. mori GAPDH* gene was used as an internal reference. Error bars represent means ± S.D. of three independent values, and the asterisks indicate significant differences compared with the control group (unpaired *t*-test; *, *p* < 0.05; **, *p* < 0.01; ns, not significant).

In order to determine the specific biological function of *BmIML-2*, we performed a qRT-PCR experiment to detect the tissue expression patterns and inducible expression patterns following viral infection. As shown in Figure 3A, *BmIML-2* exhibited the highest transcript level in fat body compared to other tissues examined. Then, 24 h after viral infection, the mRNA levels of *BmIML-2* in fat body and malpighian tube significantly increased upon BmNPV infection. In addition, the transcription level of *BmIML-2* showed a downward trend after 48 h and 72 h viral infection compared to the control group (Figure 3B,C).

### 2.3. Overexpression of BmIML-2 Inhibited BmNPV Infection and Replication

**Figure 4:**
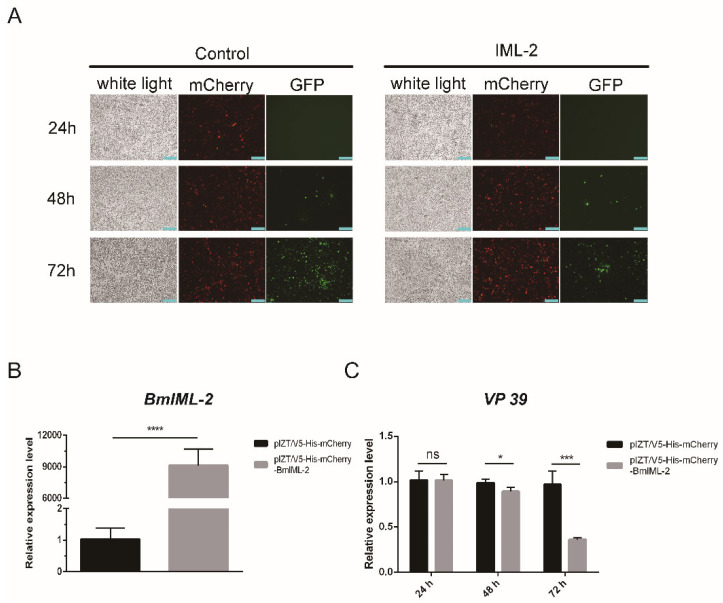
Effects of BmNPV infection and replication by overexpression of *BmIML-2* in BmN cells. (**A**) The infected cells after overexpression were observed under fluorescence microscope. White light, optical transmission; GFP, *green*; mCherry, *red*; *scale bar* = 200 µm. (**B**) Transcript level analysis of *BmIML-2* at 48 h after transfection of the overexpression vector. (**C**) Transcript level analysis of *VP 39* after overexpression of *BmIML-2* at different times. Data were calibrated by using *BmGAPDH* and presented as means ± S.D. of three separate experiments. *Asterisks* represent significant differences compared the control (unpaired *t*-test; *, *p* < 0.05; ***, *p* < 0.001; ****, *p* < 0.0001; ns, not significant).

To further explore the role of *BmIML-2* in the regulation of virus infection, we constructed the pIZT/V5-His-mCherry-BmIML-2 vector and transfected it into BmN cells. Our results suggested that the density of green fluorescence was significantly lower than that of the control group after 72 h viral infection. This indicated overexpression of *BmIML-2* dramatically inhibited the infection and replication of BmNPV. Furthermore, viral nucleocapsid protein gene *VP 39* of BmNPV was selected to determine the viral replication by using qRT-PCR assay. The results indicated that the number of BmNPV in the treatment group was dramatically decreased compared with the control group after 48 h and 72 h viral infection (Figure 4).

### 2.4. Knockdown of BmIML-2 Promoted BmNPV Infection and Replication

**Figure 5:**
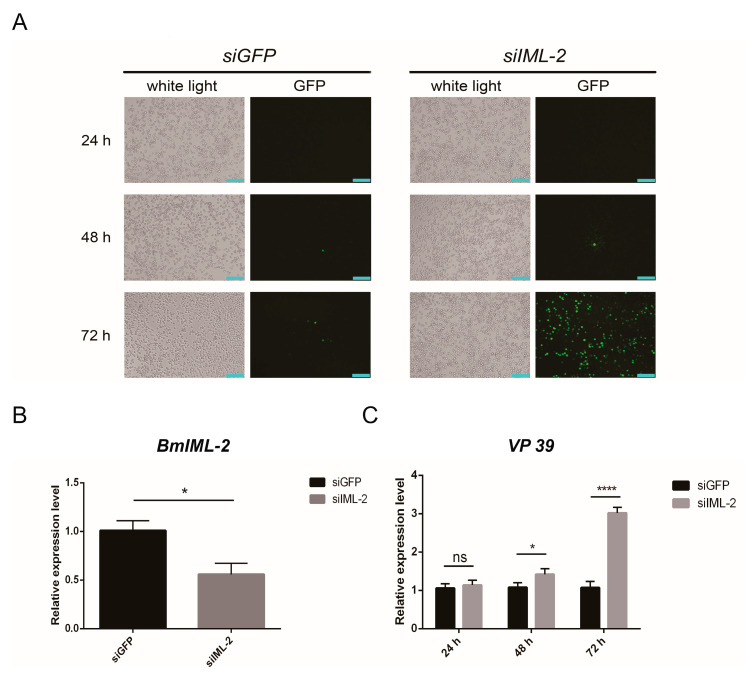
Effects of BmNPV infection and replication by knockdown of *BmIML-2* in BmN cells. (**A**) The infected cells after knockdown of *BmIML-2* were observed under a fluorescence microscope. White light, optical transmission; GFP, *green*; mCherry, *red*; *scale bar* = 200 µm. (**B**) Transcript level analysis of *BmIML-2* at 48 h after transfection of siRNA. (**C**) Transcript level analysis of *VP 39* after knockdown of *BmIML-2* at different times. Data were calibrated by using *BmGAPDH* and presented as means ± S.D. of three separate experiments. Asterisks represent significant differences compared the control (unpaired *t*-test; *, *p* < 0.05; ****, *p* < 0.0001; ns, not significant).

In order to further verify the above experimental results, the transcript level of *BmIML-2* was knocked down by using siRNA, and the replication level of BmNPV was examined. BmN cells were transfected with siRNA that was designed to target the *BmIML-*2 gene. Our result indicated that the transfection of *siIML-2* led to a significant decrease in the expression of *BmIML-2* as detected by qRT-PCR (Figure 5B). Moreover, BmN cells were infected with BmNPV-EGFP for 72 h after siRNA treatment. As shown in Figure 5C, the expression of *VP39* exhibited a marked increase at 48 h and 72 h post-infection. The results of fluorescence microscopy also confirmed that knockdown mediated by siRNA caused an increment of viral replication, especially at 72 h after infection (Figure 5A). 

### 2.5. Overexpression of BmIML-2 Affected the Expression of Apoptosis-Related Genes

**Figure 6:**
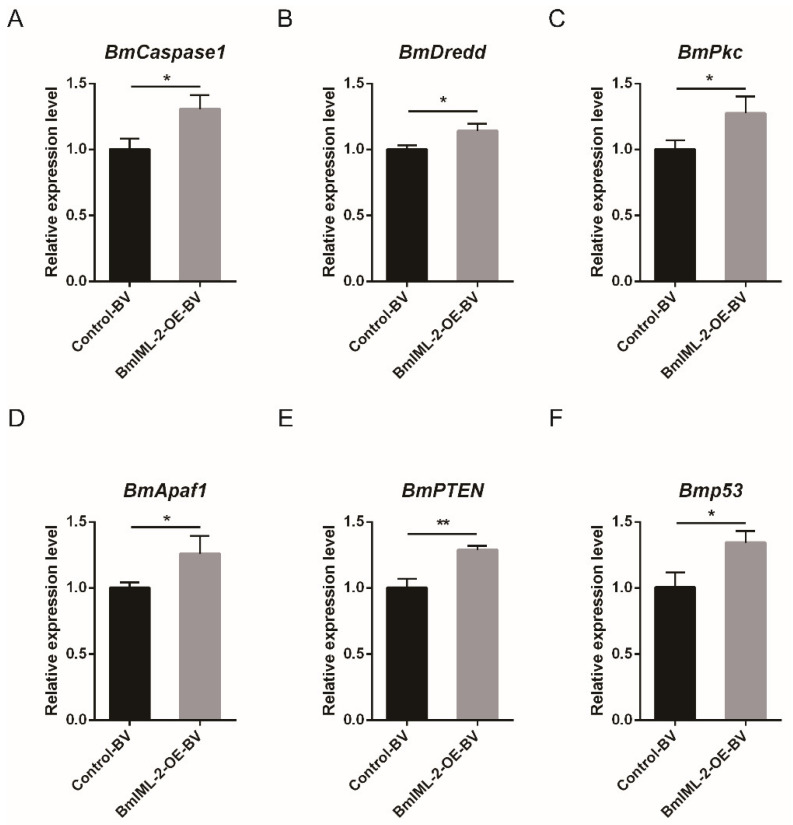
Transcription level changes of selected apoptosis-related genes in virally infected *BmIML-2* overexpressed cells (**A**–**F**). The mRNA levels of genes were detected by using qRT-PCR assay after transfection of *BmIML-2* overexpression vector. The data were calibrated by using *BmGAPDH*. Error bars represent means ± S.D. of three separate experiments. Asterisks indicate significant differences compared with the control (unpaired *t*-test; *, *p* < 0.05; **, *p* < 0.01; ns, not significant).

Firstly, the detection of *BmIML-2* transcript level after overexpression was carried out using the qRT-PCR method. The data suggested that the mRNA expression level of *BmIML-2* increased significantly in the treatment group after 48 h transfection compared with the control (Figure 4B). In order to verify whether *BmIML-2* inhibited the proliferation of BmNPV by regulating the apoptosis of BmN cells, we examined the changes of transcript level of apoptosis-related genes in the BmNPV-infected *BmIML-2*-overexpressing cells. As shown in Figure 6, qRT-PCR analysis showed that the transcript level of apoptosis-related genes including *BmCaspase1*, *BmDredd*, *BmPkc*, *BmApaf1*, *BmPTEN*, and *Bmp53* were dramatically increased in *BmIML-2* overexpressed cells following viral infection (Figure 6). Moreover, overexpression of *BmIML-2* alone without viral infection did not cause significant changes in the transcript levels of the above apoptosis-related genes (Figure S1). These results implied that *BmIML-2* may be involved in signaling pathways that regulate the expression of apoptosis-related genes, thereby modulating viral infection and replication.

### 2.6. Overexpression of BmIML-2 Facilitated BmNPV-Induced Apoptosis

**Figure 7:**
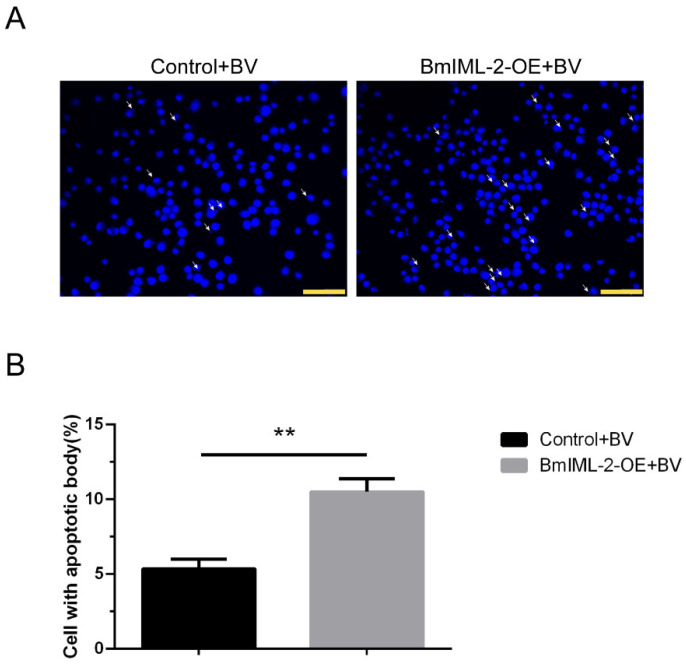
Observation of apoptotic bodies induced by BmNPV after the overexpression of *BmIML-2* in BmN cells. (**A**) The BmN cells overexpressing *BmIML-2* were infected with BmNPV. After 48 h viral infection, cells were visualized under a fluorescence microscope. Nuclei stained with 4,6-diamidino-2-phenylindole (DAPI), *blue*, *scale bar* = 100 µm. Apoptotic bodies are marked with white arrows. (**B**) The percentage of cells in apoptotic morphology. Statistical analysis was carried out by using GraphPad Prism 6.0 software. Error bars represent means ± S.D. of three separate experiments. Asterisks indicate significant differences compared with the control (unpaired *t*-test; **, *p* < 0.01).

To further confirm whether *BmIML-2* facilitates apoptosis induced by BmNPV infection, the apoptosis morphology of cells after *BmIML-2* overexpression was discriminated under an inverted fluorescence microscope. Firstly, out data suggested that almost no apoptotic bodies were observed in BmN cells that overexpressed *BmIML-2* alone without BmNPV infection (Figure S2). As shown in Figure 7A, compared with the control group transfected with empty vector, formation of abundant apoptotic bodies was evident in the virally infected *BmIML-2*-overexpressing cells. In addition, only a few apoptotic cells were observed in the control group. Moreover, percent counts of cells with apoptotic body in both groups indicated that overexpression of *BmIML-2* could significantly facilitate BmNPV-induced apoptosis in BmN cells. 

### 2.7. Immunofluorescence Localization Analysis of BmIML-2

**Figure 8:**
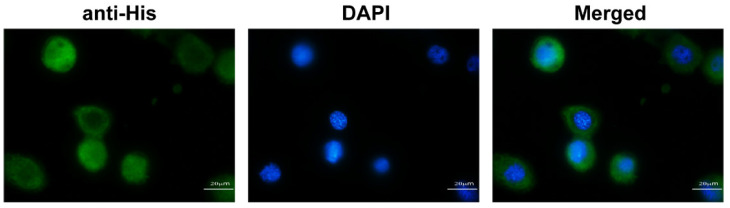
Subcellular localization of BmIML-2 in BmN cells. Cells were transfected with pIZ/V5-His-IML-2 and cultured for 48 h. Then, the cells were fixed, permeabilized, and stained. 4,6-Diamidino-2-phenylindole (DAPI) labels nuclei, shown in *blue*; Alexa-Flour-488-conjugated goat anti-mouse antibody labels BmIML-2, shown in *green*; *scale bar* represents 20 µm.

In order to determine the exact location of the protein in the cells, we detected the localization of BmIML-2 by immunofluorescence method and fluorescence microscopy. The recombinant vector pIZT/V5-His-BmIML-2 was transfected into BmN cells for transient overexpression. Then, overexpressed BmIML-2 was labeled with Alexa-Flour-488-conjugated goat anti-mouse secondary antibody, and nuclei were stained with DAPI. As shown in Figure 8, our results demonstrated that BmIML-2 evenly distributed throughout the cytoplasm and was slightly concentrated in the cell membrane. 

## 3. Discussion

BmNPV is an important infectious pathogen that endangers sericulture production, causing great economic losses to sericulture. Insect CTLs are widely known as important PRRs that contribute to triggering and regulation of host immune responses, as well as in defense against pathogens [23,24]. In the present study, we selected a putative CTL gene from the genome database of silkworm, called *BmIML-2*, to investigate its potential functions during the infection and replication of BmNPV. Our results demonstrated that *BmIML-2* could significantly inhibit the proliferation of BmNPV, which might play key roles in viral infection. Bioinformatic analysis results indicated that mature BmIML-2 contain an EPD and an EPN motif. The characteristic motif was critical for the determination of carbohydrate-binding capacity. The CTLD-containing QPD motif known as galactose-type sugar binding exhibits an affinity to galactose specifically, while CTLD with EPN motif would prefer to bind mannose-type sugar [15]. Hence, we speculated that BmIML-2 may have the ability to specifically recognize and bind to several viral envelope proteins in the early stages of BmNPV infection.

The mRNA of *BmIML-2* was detected in the selected tissues, and it exhibited the highest expression level in fat bodies, followed by malpighian tubes. Interestingly, its transcript level significantly increased at 24 h after silkworms received BmNPV infection, while it was dramatically downregulated 48 h and 72 h after viral infection compared with the control group. Previous studies suggested that the expression level of *B. mori* βGRP4 dramatically decreased from 12 h to 72 h after BmNPV infection [25]. It was likely that more BmIML-2 were induced and were secreted to recognize the invading BmNPV within 24 h of infection, and BmNPV might suppress the host’s own immune defense mechanisms after 48 h to 72 h. We considered that BmIML-2 might respond to the BmNPV infection and possess multiple functions in regulating innate immune responses.

To further confirm the involvement of *BmIML-2* in the infection and replication of BmNPV, we performed overexpression and knockdown assays to determine the roles and regulation of *BmIML-2*. Our results suggested that the overexpression of *BmIML-2* led to a significant reduction of viral proliferation in BmN cells. Conversely, the increased proliferation of BmNPV was observed after the knockdown of *BmIML-2* via siRNA. Moreover, several other PRRs identified previously have also shown that up- or downregulation of these genes would significantly modulate the proliferation of BmNPV, such as BmβGRP4 [25] and BmPGRP2-2 [14]. Thus, we conceived that BmNPV, acting as a typical PPR, was provided with the effective capacity to inhibit the infection and replication of BmNPV.

Apoptosis acts as an extremely powerful response to the infection of virus, which integrates with other innate antiviral defenses in insects, then reduces the viral proliferation and spread [26,27]. More importantly, the antiviral defense mediated by apoptosis is critical for insects lacking the vertebrate-specific acquired immune system [28]. Apoptosis signal pathway in several insect cell lines seems to be initiated following the challenge of NPVs [29]. Then, the signal of apoptosis initiated by NPV challenge ultimately stimulates the caspase cascade, which consists of upstream promoters and downstream effector caspases [30,31,32]. In silkworm, the *Bm-p53* gene was able to regulate apoptosis via the activation of caspase cascade, and overexpression of *Bm-p53* in BmN cells promoted apoptosis [33]. To further confirm whether *BmIML-2* regulates the infection and replication of BmNPV by involving the signal pathway of apoptosis, *BmIML-2* was overexpressed in the BmN cells and then infected with BmNPV to induce apoptosis. The results showed that the relative transcript level of apoptosis-related genes such as *BmCaspase1*, *BmDredd*, *BmPkc*, *BmApaf1*, *BmPTEN*, and *Bmp53* was upregulated when compared with the control group. At the same time, the expression levels of several genes that are unrelated to apoptosis did not change significantly in virally infected *BmIML-2*-overexpressing cells (Figure S3). Moreover, cells overexpressing *BmIML-2* with apoptotic body formation were observed under a microscope (Figure 7). The above results provided evidence that *BmIML-2* could positively regulate the signal pathway that induces the occurrence of apoptosis in BmN cells. PRRs in insects that are involved in the regulation of apoptosis signal pathway were reported in other investigations. For example, *Bombyx mori* peptidoglycan recognition protein BmPGRP2-2 negatively regulated the expression of BmPTEN and then inhibited the apoptosis through repressing PTEN-phosphoinositide 3-kinase (PI3K)/Akt signaling [14]. Additionally, another important PRR, BmβGRP4, could positively regulate the transcript level of *BmPTEN* and repress the expression of an apoptosis suppressor gene named *BmIAP* [25].

In conclusion, a C-type lectin gene called *BmIML-2* was characterized and identified from the genome database of *B. mori*. The significant change of *BmIML-2* transcript level was detected in fat bodies and malpighian tubes upon BmNPV challenge. Afterwards, in vitro overexpression and knockdown assays suggested that *BmIML-2* exhibited dramatical inhibitory ability for the proliferation of BmNPV. Moreover, we demonstrated that *BmIML-2* suppressed BmNPV proliferation by participating in the regulation of the expression of apoptosis-related genes. Finally, we found that BmIML-2 was mainly localized in the cytoplasm with the help of immunofluorescence analysis. These results imply that BmIML-2 plays key roles in the process of BmNPV proliferation, possibly via the apoptosis signal pathway.

## 4. Materials and Methods

### 4.1. Preparation of Silkworm, BmN Cells, and Virus

The silkworm strain p50 was provided in the Sericulture Research Institute, Jiangsu University of Science and Technology University, Zhenjiang, China. Briefly, the larvae were raised with fresh mulberry leaves under a rearing environment of 26 ± 1 °C, 70–85% relative humidity, and a 12 h light/12 h dark photoperiod. The BmN cell line from Bombyx mori ovarian was cultured in TC-100 (Livning Biological Technology Co., Ltd., Beijing) medium supplemented with 10% (*v*/*v*) fetal bovine serum (FBS) (Gibco) contained at 28 °C. The BmNPV particles used for oral infection were suspended in sterile water (1.0 × 10^7^ OB/mL). The recombinant EGFP-tagged budded virus of BmNPV was maintained in our laboratory, which used to assess the viral infection and reproduction. 

### 4.2. Bioinformatics Analysis of BmIML-2

The EXPASY (Expert Protein Analysis System) websites (http://www.expasy.org (accessed on 3 February 2022)) were used to predict the deduced amino acids sequences, molecular weight, and isoelectric point. The SignalP-5.0 server (http://www.cbs.dtu.dk/services/SignalP/ (accessed on 4 February 2022)) was used to predict the putative signal peptides. The prediction of conserved domains and motifs was performed in the SMART website (http://smart.embl-heidelberg.de/smart/set_mode.cgi (accessed on 4 February 2022)). The phylogenetic tree analysis of BmIML-2 was generated by MEGA 6.0 software with a neighbor-joining method (bootstrap = 1000 replications, Poisson model, uniform rates). 

### 4.3. RNA Extraction and cDNA Synthesis

The first day of fifth instar silkworm larvae were anesthetized on ice and dissected; then, tissue samples including heads, midguts, fat bodies, malpighian tubes, and hemolymph were collected separately. In addition, each first day of fifth instar larvae from the same batch were infected by orally fed BmNPV suspension (1.0 × 10^7^ OB/mL). After 48 h infection, the larvae were dissected, and tissues were harvested. Then, the total RNA samples from the aforementioned tissues were extracted separately with TRIzol Reagent (TIANGEN, Biotech Co., Ltd., Beijing, China). In addition, the first strand cDNA for subsequent experiments was synthesized by using a FastKing RT Kit following the manufacturer’s recommendations (TIANGEN, China).

### 4.4. Overexpression Vector Construction and Small Interfering RNA (siRNA) Synthesis

The ORF region of the *BmIML-2* was cloned using the specific primers listed in Table S1 that provided the restriction sites *Kpn* I and *Xba* I. The amplified PCR products were ligated with pMD-19T vector for sequencing verification. The digested fragment was constructed into the pIZT/V5-His-mCherry vector that had undergone the same double-digestion treatment. Then, the constructed vector was verified by double digestion and sequencing, and the empty vector was used as a control. Moreover, the siRNA targeting *BmIML-2* were synthesized by GenePharma (Suzhou, China). The oligonucleotide sequence designed for *BmIML-2* was *siIML-2* (5′-GCGCUGAUCAAUGACCUUUTT-3′), and a sequence designed for *siEGFP* (5′-GCGAUGCCACCUACGGCAATT-3′) was used as a negative control.

### 4.5. Cell Transient Transfection and BmNPV Infection

BmN cells were seeded into 12-well culture plates for 24 h and transfected with the overexpression vector or siRNA by using GP-transfect-Mate transfection reagent (GenePharma, Suzhou, China) according to the manufacturer’s instructions. Briefly, 1.6 µg overexpression vector or 80 pm siRNA oligo was added to 100 µL serum-free TC-100 medium and then mixed with an equal volume of medium containing 5 µL of transfection reagent for the preparation of transfection complexes. After incubation at room temperature for 20 min, the complex was finally added to the culture medium for transfection. Afterwards, the transfected BmN cells in each well were infected with BmNPV-EGFP with a multiplicity of infection (MOI) of 3. Then, cells were harvested 24, 48, and 72 h after viral infection, and genomic DNA or total RNA were extracted for subsequent qRT-PCR analysis. Moreover, the fluorescence intensity in the wells was observed and photographed at different points in time under an inverted fluorescence microscope (Ti-E, Nikon, Tokyo, Japan).

### 4.6. Genomic DNA Extraction

Genomic DNA of BmN cells was extracted with appropriate volume DNA extraction buffer (10 mM Tris-HCl, 100 mM EDTA, 100 mM NaCl, 0.5% SDS; pH = 8.0). After adding an equal volume of saturated phenol to mix thoroughly, we centrifuged the mixture for 15 min, aspirated the supernatant, and purified the mixture with chloroform isoamyl alcohol and absolute ethanol containing 0.1 M sodium acetate (NaAc), followed by RNase treatment. The purity and concentration of DNA were measured by a NanoDrop 2000 spectrophotometer (Thermo Fisher Scientific, NewYork, NY, USA) and were stored at −80 °C for further use.

### 4.7. Quantitative Real-Time PCR (qRT-PCR) Analysis

The transcript levels of *BmIML-2* or other genes were quantified using qRT-PCR assay. Specific primers listed in Table S1 were used. The qRT-PCR solution was prepared with UltraSYBR Mixture (ComWin Biotech Co.,Ltd., Beijing, China) according to the manufacturer’s recommendations. In addition, the qRT-PCR assay was carried out on a LightCycler^®^ 96 PCR instrument (Roche, Basel, Switzerland). The amplification conditions consisted of a pre-denaturation at 95 °C 10 min, followed by 40 additional cycles of denaturation at 95 °C for 15 s, and annealing and extension at 60 °C for 1 min. Each reaction was performed in 3 biological and 2 technical replicates. The relative expression level of each mRNA was quantified with the 2^−ΔΔCt^ method. *B. mori* glyceraldehyde-3-phosphate dehydrogenase (*BmGAPDH*) gene was used as an internal reference to calibrate the total amount of RNA. 

### 4.8. Observation of Apoptosis Morphology of Cells after BmIML-2 Overexpression

Briefly, the BmN cells overexpressing *BmIML-2* were infected with BmNPV-EGFP with a MOI of 3. After 48 h infection, cells were fixed with 4% paraformaldehyde for 20 min after culture medium was removed completely. After that, cells were washed three times with sterile PBS, and the cell nuclei were stained with 4,6-diamidino-2-phenylindole (DAPI) (Sangon Biotech Co., Ltd., Shanghai, China) (0.5 ug/mL) for 15 min. Then, the treated cells were observed by using a fluorescence microscope. The cells transfected with empty vector were used as a control.

### 4.9. Immunofluorescence Analysis of BmIML-2

Firstly, the BmN cells overexpressing *BmIML-2* (polyhistidine tag) cultured overnight for 24 h were suspended and placed on the cell round coverslip for 4 h. Then, the cells were rinsed with PBS for three times and fixed with 4% paraformaldehyde for 20 min. After being washed three times, cells were permeated with 1% Triton X-100 (PBS preparation) for 20 min at room temperature and washed with PBS as mentioned above. Then, the permeabilized cells were blocked with 5% BSA prepared by PBST for 1 h. After that, the cells were incubated with mouse monoclonal anti-polyhistidine antibody (1:800 in 5% BSA) at 4 °C overnight. After being rinsed three times, the cells were incubated with goat anti-mouse secondary antibody conjugated with Alexa Flour 488 (1:500 in 5% BSA) for 2 h at room temperature. Subsequently, cells were rinsed with PBS, and the nuclei were stained with DAPI (0.5 ug/mL) for 15 min; then, we repeated the washing above for the last time. Finally, the stained cells were mounted and photographed under a fluorescence microscope.

### 4.10. Statistical Analysis

All treatments were carried out in triplicate, and results were represented as means ± standard deviation (S.D.). Data analysis and comparison were performed by using Graph Pad Prism 6.0 software. The difference between the two groups of data was compared with Student’s *t-test*, while analysis of variance (ANOVA) was used to test the significance of the difference between the means of two or more samples. A significant difference was considered when *p*-value < 0.05.

## Data Availability

Not applicable.

## Supplementary Materials

The supporting information can be downloaded at: https://www.mdpi.com/1422-0067/23/15/8369/s1.
