# Correction: Mei et al. *Bombyx mori* C-Type Lectin (BmIML-2) Inhibits the Proliferation of *B. mori* Nucleopolyhedrovirus (BmNPV) through Involvement in Apoptosis. *Int. J. Mol. Sci.* 2022, *23*, 8369

**DOI:** 10.3390/ijms24032224

**Published:** 2023-01-22

**Authors:** Xianghan Mei, Chun Li, Peilin Peng, Jue Wang, Enxi He, Zhiyong Qiu, Dingguo Xia, Qiaoling Zhao, Dongxu Shen

**Affiliations:** 1 Jiangsu Key Laboratory of Sericultural Biology and Biotechnology, School of Biotechnology, Jiangsu University of Science and Technology, Zhenjiang 212018, China; 2 Key Laboratory of Silkworm and Mulberry Genetic Improvement, Ministry of Agriculture and Rural Affairs, Sericultural Research Institute, Chinese Academy of Agricultural Sciences, Zhenjiang 212018, China

In the original publication [1], there was a mistake in Figure 5: Effects of BmNPV infection and replication by knockdown of BmIML-2 in BmN cells as published. Accidentally, Figure 5A–C were separately swapped for Figure 4A–C during the final corrections in the original version. The corrected Figure 5: Knockdown-IML-2 appears below. It is worth noting that Figure 5A–C in the new Figure 5 have been corrected. The authors state that the scientific conclusions are unaffected. This correction was approved by the Academic Editor. The original publication has also been updated.

## Figures and Tables

**Figure 5:**
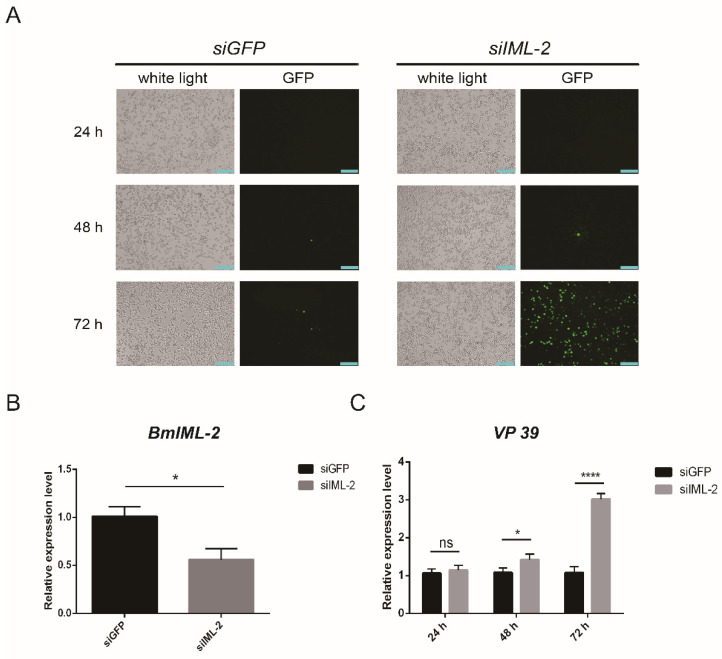
Effects of BmNPV infection and replication by knockdown of *BmIML-2* in BmN cells. (**A**) The infected cells after knockdown of *BmIML-2* were observed under a fluorescence microscope. White light, optical transmission; GFP, *green*; mCherry, *red*; *scale bar* = 200 µm. (**B**) Transcript level analysis of *BmIML-2* at 48 h after transfection of siRNA. (**C**) Transcript level analysis of *VP 39* after knockdown of *BmIML-2* at different times. Data were calibrated by using *BmGAPDH* and presented as means *±* S.D. of three separate experiments. Asterisks represent significant differences compared the control (unpaired *t*-test; *, *p* < 0.05; ****, *p* < 0.0001; ns, not significant).
