# Supplementary material for: Bombyx mori C-Type Lectin (BmIML-2) Inhibits the Proliferation of B. mori Nucleopolyhedrovirus (BmNPV) through Involvement in Apoptosis
Source: Int J Mol Sci. 2022 Jul 28;23(15):8369. doi: 10.3390/ijms23158369 (PMC9369074; doi:10.3390/ijms23158369)
Supplement: Supplementary file 1 [file ijms-23-08369-s001.zip › ijms-1816670-supplementary/ijms-1816670-supplementary/Supplementary Files-7-28/Figure legends of supplementary files.pdf]

**Table S1. Oligonucleotides used for plasmid construction and qRT-PCR.**

**Figure. S1**. **Expression levels changes of selected apoptosis-related genes in *BmIML-2* overexpressed cells.** The mRNA levels of genes were detected using qRT-PCR after transfection of *BmIML-2* overexpression vector. The *BmGAPDH* used as a housekeeping gene. Error bars represent means ± S.D. of three separate experiments.

**Figure. S2**. **Observation of nuclei after the overexpression of *BmIML-2* in BmN cells without viral infection.** The BmN cells were transferred with *BmIML-2* overexpression vector. The empty vector transfected cells were used as a control. After 48 h transfection, cells were visualized under a fluorescence microscope. Nuclei stained with 4,6-Diamidino-2-phenylindole (DAPI), *blue*. Apoptotic bodies are marked with white arrows.

**Figure. S3**. **Transcription level changes of selected apoptosis-unrelated genes in viral-infected *BmIML-2* overexpressed cells.** The mRNA levels of genes were quantified using qRT-PCR after transfection of *BmIML-2* overexpression vector. The data was normalized by using *BmGAPDH*. Error bars represent means ± S.D. of three separate experiments.
