# Supplementary material for: Bombyx mori C-Type Lectin (BmIML-2) Inhibits the Proliferation of B. mori Nucleopolyhedrovirus (BmNPV) through Involvement in Apoptosis
Source: Int J Mol Sci. 2022 Jul 28;23(15):8369. doi: 10.3390/ijms23158369 (PMC9369074; doi:10.3390/ijms23158369)
Supplement: Supplementary file 1 [file ijms-23-08369-s001.zip › ijms-1816670-supplementary/ijms-1816670-supplementary/Supplementary Files-7-28/Table S1-BmIML-2 Primer sequences.pdf]

Table S1. Oligonucleotides used for plasmid construction and qRT-PCR.

	Forward Primers	Reverse Primers
Amplification of entire coding sequences of IML-2:		
	5'-ATGAAAGCGGCGAATAAAAGTC-3'	5'-TCATTTGTCGTCGTGCTCTCGGA-3'
qRT-PCR:		
IML-2	5'-AGAGTTCGTGTGGCATCTA-3'	5'-AGAGTTCGTGTGGCATCTA-3'
GAPDH	5'-TTCATGCCACAACTGCTACA-3'	5'-AGTCAGCTTGCCATTAAGAG-3'
VP39	5'-CAACTTTTTGCGAAACGACTT-3'	5'-CAACTTTTTGCGAAACGACTT-3'
Caspase1	5'-GGAAAAACGGCAATGAAGAC-3'	5'-AACACAGCAACCAGCAGACA-3'
Dredd	5'-TAATAGTCGTTCTGACTTGGGACA-3'	5'-TCGGTATGCAATGCAGTTTCT-3'
Pkc	5'-TGCTCTACCCCGTGTGGC-3'	5'-TCCTGGTTGATGGTCCGC-3'
Apaf1	5'-ACTGCAAAGTCTCAACCATA-3'	5'-CAAAGCACAACATTACCACA-3'
PTEN	5'-CTGATAGTGGAGAAGGTGCCG-3'	5'-GTAATGGCCGACGCGCT-3'
p53	5'-CGCCTTCAAGTTCGTCTG-3'	5'-TTCACCGTTTTGGCTCCG-3'
Amplification of mature IML-2, excluding signal peptide, with restriction sites for cloning in pIZT/V5-His-mCherry vector:		
	5'-TAGGTACC**ATG**AAAGCGGCGAATAAAAGTC-3'	*Kpn I*
	5'-GCTCTAGACCTTTGTCGTCGTGCTCTCGGA-3'	*Xba I*
	Added *Kpn I* site is underlined.	
	Added *Xba I* site is underlined.	
	Start codon is bolded.	
