# Supplementary figures and images for: Bombyx mori C-Type Lectin (BmIML-2) Inhibits the Proliferation of B. mori Nucleopolyhedrovirus (BmNPV) through Involvement in Apoptosis
Source: Int J Mol Sci. 2022 Jul 28;23(15):8369. doi: 10.3390/ijms23158369 (PMC9369074; doi:10.3390/ijms23158369)

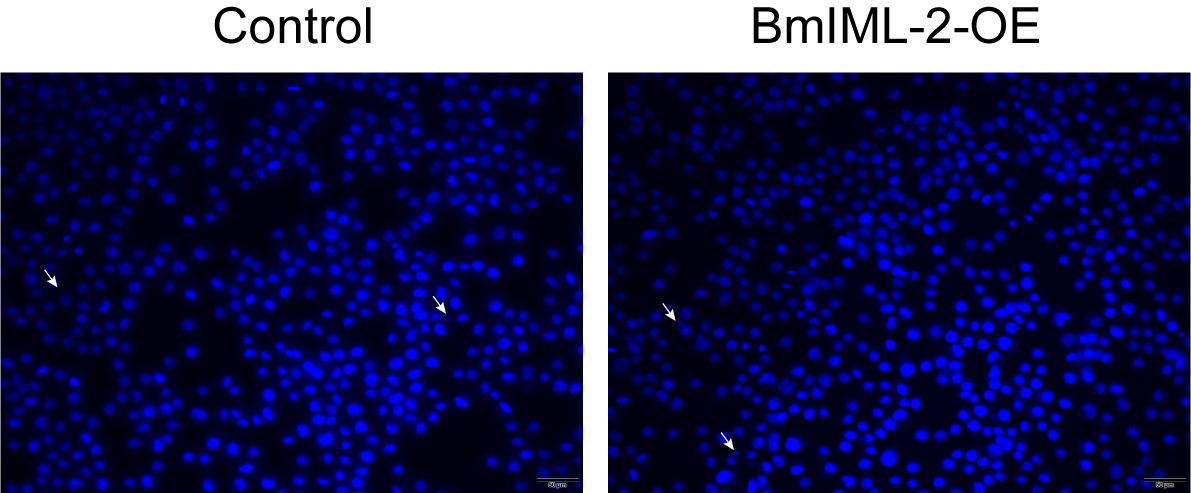

Supplement: Supplementary file 1 [file ijms-23-08369-s001.zip › ijms-1816670-supplementary/ijms-1816670-supplementary/Supplementary Files-7-28/Figure S2 Observation of apoptosis without infection.tif]

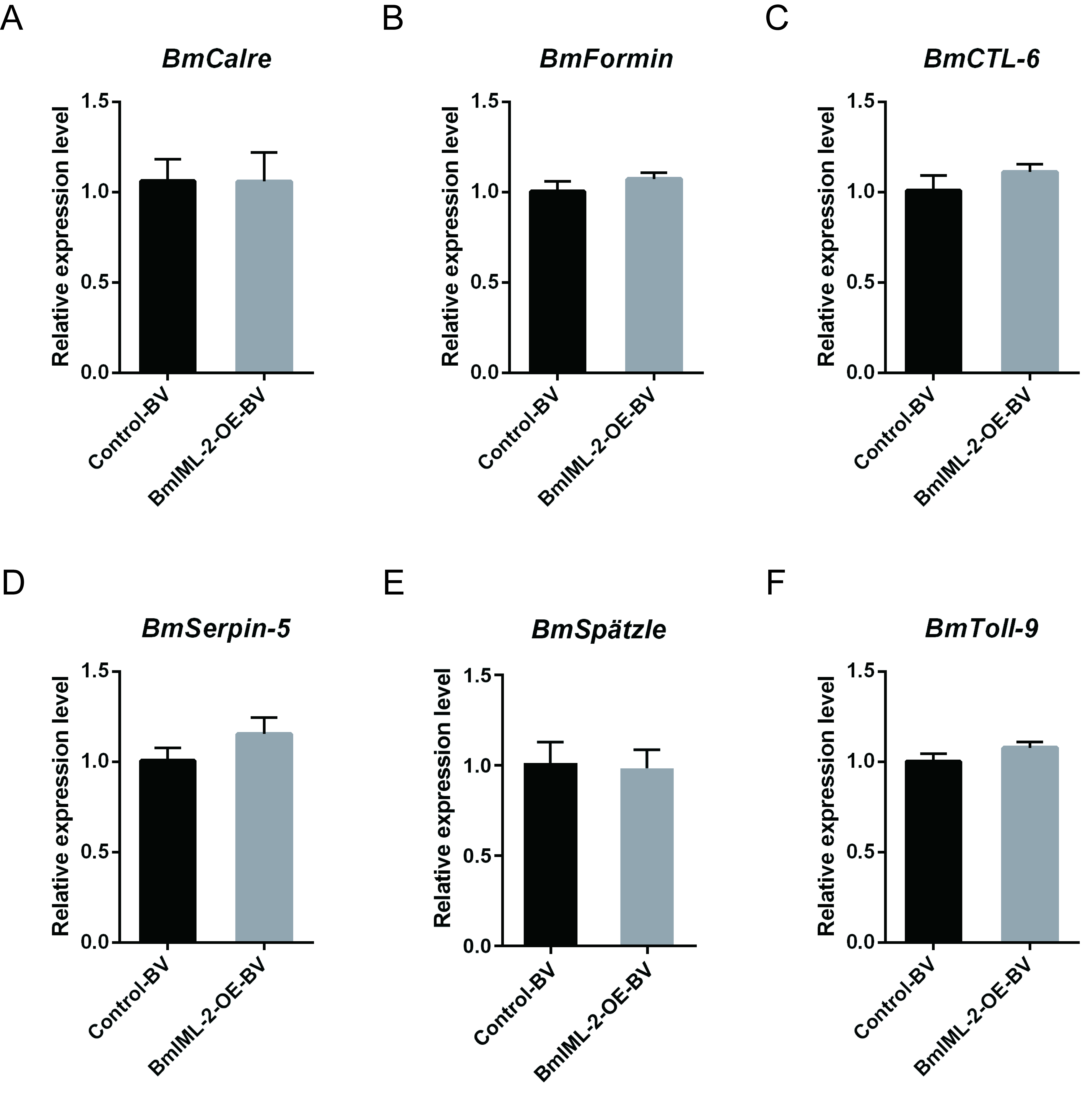

Supplement: Supplementary file 1 [file ijms-23-08369-s001.zip › ijms-1816670-supplementary/ijms-1816670-supplementary/Supplementary Files-7-28/Figure S3 other genes detection-IML-2.tif]
